# Classification of Children's Heart Sounds With Noise Reduction Based on Variational Modal Decomposition

**DOI:** 10.3389/fmedt.2022.854382

**Published:** 2022-05-26

**Authors:** Anqi Zhang, Jiaming Wang, Fei Qu, Zhaoming He

**Affiliations:** ^1^Research Center of Fluid Machinery Engineering and Technology, Jiangsu University, Zhenjiang, China; ^2^Nanjing Drum Tower Hospital, The Affiliated Hospital of Nanjing University Medical School, Nanjing, China; ^3^Shanghai Lishen Information Technology Co., Ltd., Shanghai, China; ^4^Department of Mechanical Engineering, Texas Tech University, Lubbock, TX, United States

**Keywords:** congenital heart disease, wavelet soft threshold, variational mode decomposition, heart sound denoising, intelligent classification

## Abstract

**Purpose:**

Children's heart sounds were denoised to improve the performance of the intelligent diagnosis.

**Methods:**

A combined noise reduction method based on variational modal decomposition (VMD) and wavelet soft threshold algorithm (WST) was proposed, and used to denoise 103 phonocardiogram samples. Features were extracted after denoising and employed for an intelligent diagnosis model to verify the effect of the denoising method.

**Results:**

The noise in children's phonocardiograms, especially crying noise, was suppressed. The signal-to-noise ratio obtained by the method for normal heart sounds was 14.69 dB at 5 dB Gaussian noise, which was higher than that obtained by WST only and the other VMD denoising method. Intelligent classification showed that the accuracy, sensitivity and specificity of the classification system for congenital heart diseases were 92.23, 92.42, and 91.89%, respectively and better than those with WST only.

**Conclusion:**

The proposed noise reduction method effectively eliminates noise in children's phonocardiograms and improves the performance of intelligent screening for the children with congenital heart diseases.

## Introduction

The prevalence of congenital heart diseases among newborns in China is rising up to 0.898% (1, 2). Among them, ventricular or atrial septal defects are the most common. Pathological murmurs are produced by blood flow through the abnormal cardiovascular morphology and structure in addition to the periodic first or second heart sounds (3–5). Therefore, heart auscultation is the main method of screening for congenital heart diseases (6). Phonocardiogram (PCG) is generated by the use of an electronic stethoscope, which promotes intelligent diagnosis for congenital heart diseases (7), in which time domain features of heart sounds (8), frequency domain features (9, 10) and time-frequency domain features (11, 12) have been analyzed in the machine learning models (11, 13) or neural networks (12, 14). However, PCG often contains noise due to the subject ambient and activity conditions, such as power noise, breathing sounds, friction between the piezoelectric thin-film sensor of the stethoscope and the body surface, and especially for children whose emotions are uncontrolled. Young children are prone to struggle and cry, and their PCG is relatively weak with a low signal-noise ratio. Noise in the recordings mixes randomly and intermittently with heart sounds, or may exhibit bandwidth characteristics, or pulsatility, all of which can reduce the accuracy of PCG feature analysis and extraction. Therefore, it is necessary to effectively reduce the noise of children's PCG from different sources and manifestations before feature extraction for the intelligent diagnosis of the congenital heart disease.

Currently, denoising methods for PCG are mainly divided into 3 categories: blind source separation algorithms (15–17), adaptive noise reduction based on empirical mode decomposition (18), and threshold noise reduction based on wavelet (10, 11, 14, 19). The blind source separation algorithms, relying on information theory and matrix analysis, are often used to distinguish heart and lung sounds and to denoise them. The matrix decomposition algorithms, which are typical blind source separation algorithms, such as singular value decomposition (16) and non-negative matrix decomposition (15), are employed to restore or isolate the sound source. However, blind source separation algorithms lead to complex and tedious operation and do not deal with the difference between the noise and murmur. It is unknown whether the blind source separation algorithm scan distinguishes the noise from abnormal heart or lung sounds. The original murmur part could be lost after the PCG from ventricular septal defect patients were denoised with 2D group sparsity algorithm (17). The denoised signal was only used for heart sound localization, but not for murmur extraction. The empirical mode decomposition which is a spatiotemporal filter (18, 20) decomposes the PCG non-linearly in the time domain (21, 22) with good adaptability, but has problems such as modal aliasing and end effect. The problems remain unsolved even if the improvements were proposed (23, 24). The wavelet threshold method is a common noise reduction method in the heart sound classification (10, 11, 14) where the difference in thresholds of detailed components of each scale between the signal and noise under orthogonal wavelet transformexists to reconstruct the denoised signal. However, wavelet denoising method lacks self-adaptability and has poor suppression effect on the burst noise. The VMD algorithm proposed by Dragomiretskity (25) based on the variational theory in functional analysis can overcome the end effect and modal aliasing problems of the empirical mode decomposition, and has stronger noise robustness. Recently, the VMD method has achieved good results in the field of vibration signal noise reduction (26, 27). It has also been employed for PCG segmentation of the first and second heart sounds (17, 28), but rarely for noise reduction (29). Although VMD is a well-established signal processing method, there are still some shortcomings in its application for PCG denoising. First, the modes cannot be flexibly selected according to the actual decomposition of PCG under various noise intensities (29). Second, the decomposition performance of VMD decreases with the increase in noise intensity (26, 27), resulting in the residual noise in some of the modes that contain heart sound information. When the number of decomposition layers and the range of modalities used for signal reconstruction are fixed, the denoising performance of VMD significantly decreases under strong Gaussian noise. Therefore, neither VMD nor WST can effectively reduce strong ambient noise or the burst noise in children's PCGs under non-standard acquisition environments. Therefore, we aimed to develop a noise reduction method combining both VMD and WST to improve intelligent diagnosis of children's congenital heart diseases.

## Materials and Methods

### PCG Recording

**Figure 1:**
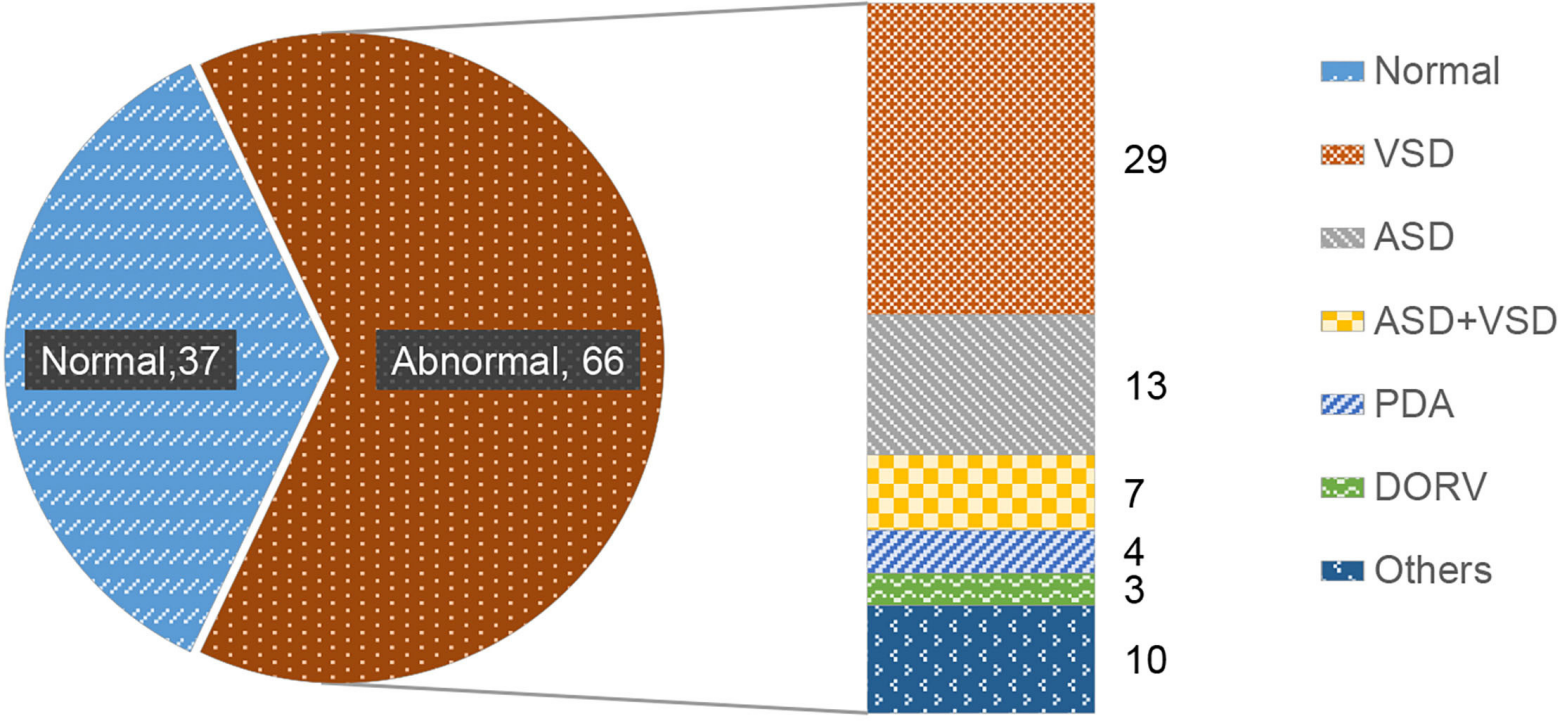
Distribution of heart sound types. Normal heart sound (normal), ventricular septal defect (VSD), atrial septal defect (ASD), atrial septal defect + ventricular septal defect (ASD + VSD), patent ductus arteriosus (PDA), right ventricular double outlet (DORV), other pathological murmurs (others).

The PCG signals of 103 subjects were obtained for the screening study by an electronic stethoscope (ChildCare G-100, Shanghai Tuoxiao Intelligent Technology Co., Ltd., Shanghai, China) in a standard clinical setting with a hardware sampling rate of 44.1 KHz and 16 bit AD sampling. The demography of 103 samples was shown in Figure 1 with 37 normal heart sounds and 66 pathological precordial murmurs. All the subjects have signed the informed consent form with the hospitals. All PCG signals were resampled to 2,000 Hz and processed with MATLAB 2020a.

### Principle of VMD

VMD defines the Intrinsic Mode Functions (IMFs) as Equation (2.1).


(2.1) $$\begin{array}{r} u_{k}\left(t\right)=A_{k}\left(t\right)\cos\left(\phi_{k}\left(t\right)\right) \end{array}$$

Where *u*_*k*_(*t*) is an amplitude-modulated-frequency-modulated signals that represents the k-th IMF, ϕ_*k*_(*t*)I is a non-decreasing function, ${\phi_{k}}^{{}^{\prime }}\left(t\right)\geq 0$. *A*_*k*_(*t*) is slowly varying with respect to ϕ_*k*_(*t*).

VMD aims to decompose signal *x*(*t*) into *K* modes with limited bandwidth. Hilbert transform for *u*_*k*_(*t*) exist to obtain 1-sided spectrum. The 1-sided spectrum is modulated to the fundamental band by multiplication with the complex exponential. Then, the bandwidth is estimated by the squared *L*^2^ of the gradient of the demodulated signal. Correspondingly, the constrained variational model is expressed as:


(2.2) $$\begin{array}{r} \min_{\left\{u_{k}\right\},\left\{\omega_{k}\right\}}\left\{\underset{k}{\sum }||\partial_{t}\left[\delta \left(t\right)+\frac{j}{\pi t}u_{k}\left(t\right)\right]e^{-j\omega_{k}t}|{|}_{2}^{2}\right\}\\ s.t.\underset{k}{\sum }u_{k}=x \end{array}$$

Where, ω_*k*_ is center frequency corresponding to *u*_*k*_.Quadratic penalty factors α and Lagrange multipliers λ are applied to solve the constrained model. Augmented Lagrange multipliers are combined with the operator alternating direction method to iteratively solve ω_*k*_ and *u*_*k*_ for well convergence property of the quadratic penalty under finite weights and strict enforcement of the constraints:


(2.3) $$\begin{array}{r} L\left(\left\{u_{k}\right\},\left\{\omega_{k}\right\},\lambda \right)\\ \;=\alpha \underset{k}{\sum }{\left||\partial_{t}\left[\left(\delta \left(t\right)+\frac{j}{\pi t}\right)u_{k}\left(t\right)\right]e^{-j\omega_{k}t}|\right|}_{2}^{2}\\ \;+||f\left(t\right)-\underset{k}{\sum }u_{k}\left(t\right)|{|}_{2}^{2}\\ \;+\langle \lambda \left(t\right),x\left(t\right)-\underset{k}{\sum }u_{k}\left(t\right)\rangle \end{array}$$

#### VMD Algorithm and Its Parameter Selection

1) Initialization: ${\hat{u}}_{k}^{1},\omega_{k}^{1},{\hat{\lambda }}^{1},n\leftarrow \;0$
2) Iteration: *n*←*n*+1
3) Circulation: *k* = 1:*K*, ω≥ 0

$${\hat{u}}_{k}^{n+1}\left(\omega \right)\leftarrow \frac{\hat{x}\left(\omega \right)-\underset{i<k}{\sum }{\hat{u}}_{i}^{n+1}\left(\omega \right)-\underset{i>k}{\sum }{\hat{u}}_{i}^{n}\left(\omega \right)+\frac{{\hat{\lambda }}^{n}\left(\omega \right)}{2}}{1+2\alpha {\left(\omega -\omega_{k}^{n}\right)}^{2}},$$

$$\omega_{k}^{n+1}\leftarrow \frac{\overset{\infty }{\underset{0}{\int }}\omega {\left|{\hat{u}}_{k}^{n+1}\left(\omega \right)\right|}^{2}d\omega }{\overset{\infty }{\underset{0}{\int }}{\left|{\hat{u}}_{k}^{n+1}\left(\omega \right)\right|}^{2}d\omega }$$

4) When decomposition number reaches *K*, stop the inner loop and update λ:

$$\begin{array}{l} {\hat{\lambda }}^{n+1}\left(\omega \right)\leftarrow {\hat{\lambda }}^{n}\left(\omega \right)+\tau \left(\hat{x}\left(\omega \right)-\underset{k}{\sum }{\hat{u}}_{k}^{n+1}\left(\omega \right)\right) \end{array}$$

5) Stop the iteration if the stop condition is met; otherwise, go to step 2) to continue the iteration.

$$\begin{array}{l} \underset{k}{\sum }{\left||{\hat{u}}_{k}^{n+1}-{\hat{u}}_{k}^{n}|\right|}_{2}^{2}/||{\hat{u}}_{k}^{n}|{|}_{2}^{2}<\varepsilon \end{array}$$

**Figure 2:**
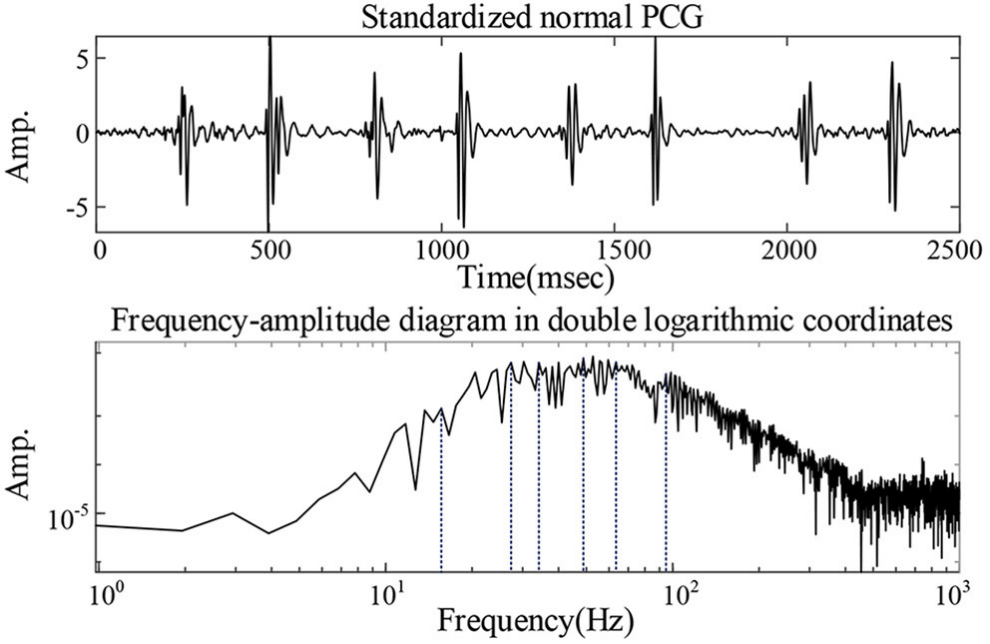
Analysis of the value of *K*: the frequency-amplitude in double logarithmic coordinates of heart sound of a 1-year-old healthy boy collected in a quiet environment.

Where τ is the Lagrangian multiplier update parameter, also known as the noise tolerance, and was set to 0. The penalty factor α was set to 2,500 (17). The *K*-value was set by analyzing the frequency-amplitude spectrum of the heart sound signal. The frequency-amplitude in double logarithmic coordinates of heart sound of a 1-year-old healthy boy collected in a quiet environment was recorded in Figure 2. Ideally, if the frequency-amplitude curve of the signal has *n* peaks, *K* = *n*. However, excessive decomposition layers are likely to cause overlapping frequencies of the modes. Therefore, similar wave crests were neglected in the current study and the finally *K* = 6.

### Reconstruction Modal Screening Index

Permutation entropy *pec*_*k*_ and correlation coefficient *RSig*_*k*_ were combined to establish screening indexes for reconstruction to avoid the loss of pathological information in children's heart sound signals by noise reduction processing:


(4) $$\begin{array}{r} RSp_{k}=\beta \left(1-RSig_{k}\right)+\left(1-\beta \right)pec_{k} \end{array}$$

The screening index *RSp*_*k*_ existed to find out the IMFs with low correlation coefficients and high permutation entropies for rejection. Where β is a propensity parameter set to 0.3, indicating that the mode with the higher permutation entropy was preferred. The degree of randomness of noise level is positively correlated with *pec*_*k*_. The smaller *RSig*_*k*_ is, the more random the IMF is.

### Reconfiguration Process

After decomposing the PCG into 6 layers of VMD, the *RSp*_*k*_ of each IMF was calculated. The IMFs with *RSp*_*k*_ greater than the index threshold *T_RSp*_*k*_ were identified as containing higher noise. In the current study, *T_RSp*_*k*_ = 0.6 was used. If the *RSp*_*k*_ of a mode was >0.6 and its *RSig*_*k*_ was <0.5, it would be rejected. These unremoved IMFs were combined with WST to obtain the denoised heart sound signal. There were 2 methods for combination with WST. Of the two methods, 1 was to denoise each IMF retained separately using WST before reconstructing and is defined as VWG. The other was to reconstruct the modalities first, and further use WST to obtain the final noise-reduced heart sound signal and is defined as VGW. Daubechies 6 wavelet was chosen, the number of decomposition layers was 6, and the threshold value was chosen to be a universal threshold.

The proposed denoising method for children's PCG can be summarized as follows.

1. Decomposition of PCG by VMD.
2. Screening the modes according to their permutation entropies and correlation coefficients with the original signal.
3. Processing the retained modes: applying WST on each mode and then reconstructing the modes; or reconstructing these modes first and then applying WST on the reconstructed signal.

### Noise Reduction Experiment

Random noise from outside the body may form an overall Gaussian ambient noise in PCG (30). To simulate the different levels ambient noise, the high-quality children's PCG signals collected by professional medical staff were superimposed with 5 and 10 dB of Gaussian white noise. The noise-added signals were then denoised with the VWG, VGW, and WST method. The VMD-based denoising method proposed in (29) was also employed for Gaussian noise reduction, where the last IMF in the method was chosen as the noise reduction signal after VMD decomposition. Signal-to-noise ratio and root mean square error help to quantitatively evaluate the denoising performance.

PCG signals of a 28-day-old child with congenital heart disease were also employed for experiment. The child was suffering from ventricular septal defect (1.0 cm from perimembranous to sublet valve), a trial septal defect (ostium secundum is 0.2 cm), mild tricuspid regurgitation, slightly stronger mitral valve cusp echo, mild to moderate mitral regurgitation, and pulmonary hypertension. The PCG was recordedunder the state of intermittent crying and about 40 s, whose noise showed intermittency in time and the intensity varied with the strength of the cry. The signal noise reduction ratio (31) existed to evaluate the noise reduction:


(5) $$\begin{array}{r} dnSNR=10*\log10\left(P_{s}/P_{d}\right) \end{array}$$

Where *P*_*s*_ is the power of the noise-containing PCG, and *P*_*d*_ is the power of the denoised PCG. The small *dnSNR* indicates that the noise is reduced and the signal is smooth. Further, the Mel filter energy of PCG recorded under severe crying was analyzed because of wide use Mel-frequency cepstral coefficients the intelligent auscultation (7, 11), where the upper and lower limit frequencies were set to 30 and 500 Hz, respectively, due to the electronic stethoscope with hardware filtering bandwidth of 30–500 Hz.

### Intelligent Diagnosis Model

PCG features were extracted after denoising, and employed for established classification model. The previously studied method of children's intelligent heart sound diagnosis (14) was adopted. A double qualification of peak detection plus threshold range was applied to the signal reconstructed after the signal was reconstructed by Hadamard product in the fourth and fifth level of detail. The types of features employed are shown in Table 1. A 10-10-1 back propagation neural network was employed for classification. The outputs of the network were quantified as 0 or 1 by a threshold of 0.5, which represent normal heart sounds or abnormal heart sounds, respectively. The Jack-Knife method (32) existed to evaluate the generalization ability of the classification system due to the limitation of the number of samples. One sample at a time from 103 cases was set aside for validation of the trained model, and the remaining 102 samples were used for training the model.

**Table 1:** The time and frequency features in closed atrioventricular valve (CAV) and closed semilunar valve (CSV) periods.

**Features**	**Description**
1–3	Max, Min, and Mean absolute in CAV
4–6	Max, Min, and Mean absolute in CSV
7–8	Max and Mean: power spectral density of CAV
9–10	Max and Mean: power spectral density of CSV

## Results

### Noise Reduction Effect Onambient Noise

**Figure 3:**
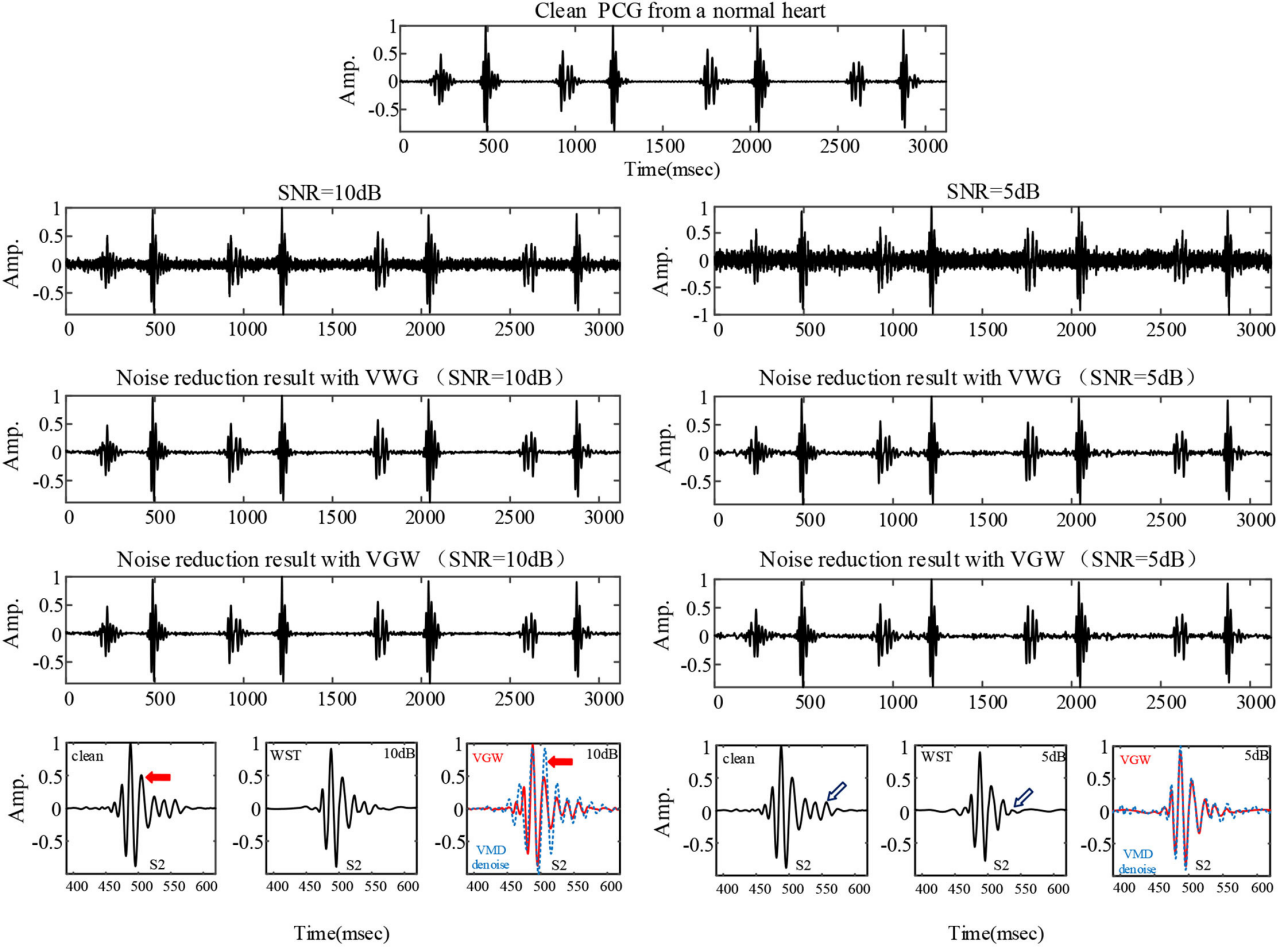
The PCG of a healthy child collected professionally in a quiet environment and the denoising results for the PCG with 5 dB and 10 dB Gaussian noise added, respectively. The comparison of details is shown at the bottom: clean PCG and denoised PCG using the WST, VGW (red line), VMD-based method (blue line).

The noise reduction effect on the simulatedambient noise added to a normal PCG is shown as an example Figure 3. From the overall waveform, the Gaussian noise with a signal-to-noise ratio of 10 dB was effectively suppressed, and there was no significant difference between the denoised PCG and the clean normal PCG. It was true also for the Gaussian noise with a signal-to-noise ratio of 5 dB but with more burrs in the baseline compared with the clean normal PCG, which meant that some noise remained. The details of the waveform containing the second heart sound are located at the bottom of Figure 3. The waveform of the second heart sound was distorted after being denoised with the VMD-based method (29). The waveform other than the second heart sound has more obvious noise fluctuations than that using the VGW when the noise intensity is enhanced. In addition, some details were lost after denoising with the WST only (marked by blue arrows). The signal-to-noise ratios and root mean square errors obtained by the simulated noise reduction for the several common pediatric PCG are shown in Tables 2, 3, respectively. The signal-to-noise ratios and root mean square errors obtained by the combined denoising method were greater than those by the WST only and the VMD-based method at the 2 different noise levels. As the noise intensity increased, the signal-to-noise ratios obtained by the VWG, VGW, and only WST decreased, and the root mean square errors increased. Generally, the difference between the signal-to-noise ratios obtained by VWG and VGW was little, except for the noise reduction of the PCG of the case of the patent ductus arteriosus. Only 4 groups of the denoising metrics obtained by the VMD-based method (29) were better and the rest groups were significantly worse than those obtained by WST.

**Table 2:** Comparison of signal-to-noise ratio (SNR) of PCG signals with Gaussian noise after noise reduction.

**PCG**	**5 dB**				**10 dB**			
	**VWG**	**VGW**	**WST**	**VMD-based**	**VWG**	**VGW**	**WST**	**VMD-based**
Normal (13Y)	15.61	15.85	9.38	13.06	19.32	19.42	12.61	3.13
Normal (4Y)	13.92	13.88	8.55	4.63	16.43	16.74	11.68	3.82
Normal (1Y)	13.30	13.55	9.88	11.65	17.28	17.43	13.34	7.68
Normal (7Y)	14.76	15.46	10.20	14.38	19.69	19.86	13.67	4.94
VSD (2Y)	8.81	8.22	4.13	7.25	9.19	9.49	6.27	2.17
ASD (4Y)	11.49	11.27	6.45	4.08	13.71	13.68	9.53	3.60
ECD (4Y)	12.86	12.83	6.59	4.69	13.92	13.77	9.58	4.67
PDA (6Y)	6.75	8.02	5.30	4.09	7.37	8.89	8.40	2.52

**Table 3:** Comparison of root mean square error (RMSE) of PCG signals with Gaussian noise after noise reduction.

**PCG**	**5 dB**				**10 dB**			
	**VWG**	**VGW**	**WST**	**VMD-based**	**VWG**	**VGW**	**WST**	**VMD-based**
Normal (13Y)	0.0266	0.0269	0.0531	0.0317	0.0145	0.0146	0.0318	0.0996
Normal (4Y)	0.0277	0.0286	0.0486	0.0735	0.0157	0.0156	0.0315	0.0807
Normal (1Y)	0.0314	0.0315	0.0475	0.0380	0.0187	0.0183	0.0311	0.0599
Normal (7Y)	0.0274	0.0275	0.0483	0.0287	0.0152	0.0151	0.0307	0.0849
VSD (2Y)	0.0467	0.0465	0.0769	0.0518	0.0377	0.0364	0.0550	0.0931
ASD (4Y)	0.0454	0.0509	0.0823	0.1049	0.0377	0.0383	0.0573	0.1109
ECD (4Y)	0.0387	0.0379	0.0756	0.0950	0.0373	0.0359	0.0547	0.0953
PDA (6Y)	0.0431	0.0350	0.0512	0.0581	0.0403	0.0353	0.0359	0.0697

**Figure 4:**
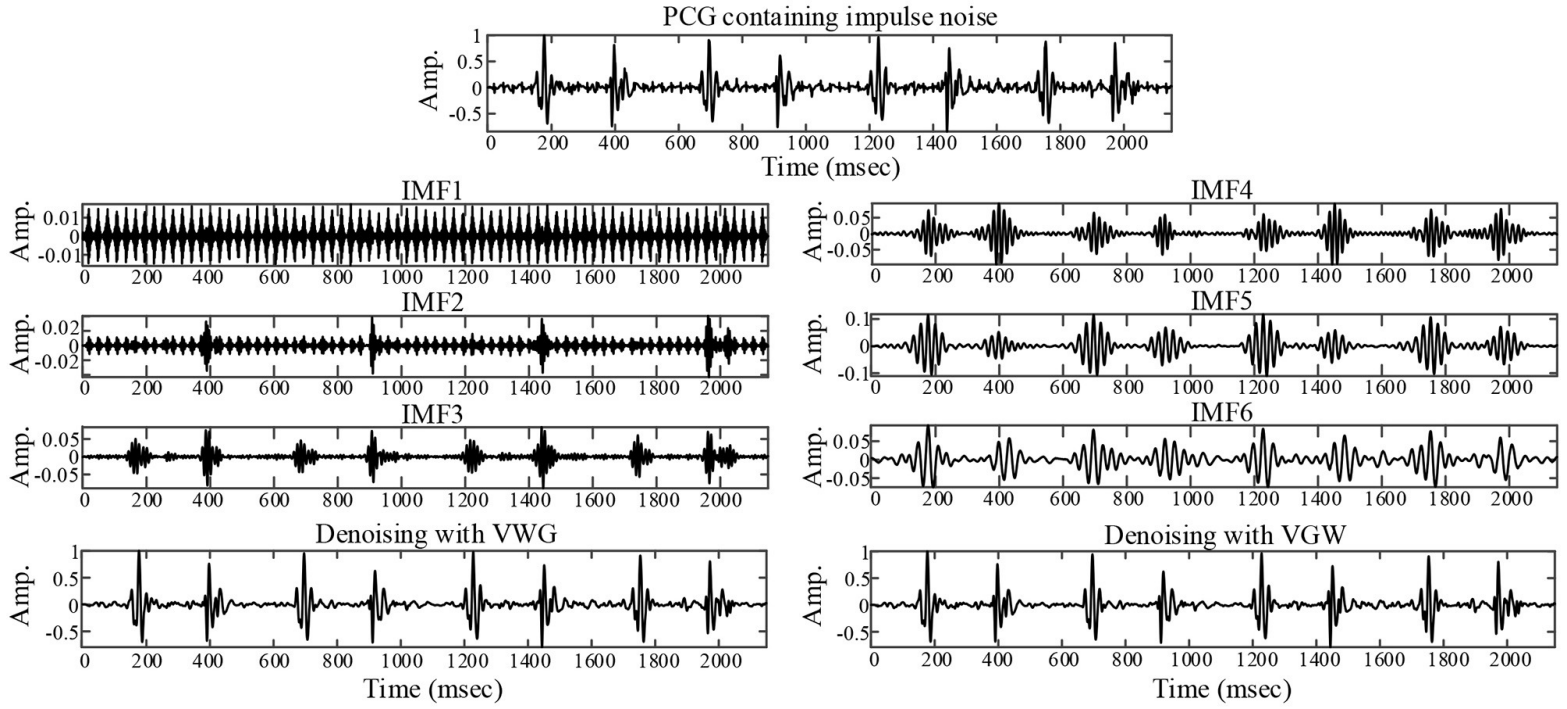
Noise reduction for a collected PCG with ambient noise that exhibits impulsive interference.

The actual ambient noise differed from the ideal analog noise due to the electronic stethoscope's hardware filtering. The noise reduction of the PCG of a healthy newborn containing periodic impulse noise is illustrated in Figure 4. The first and second IMFs containing noise components were removed by filtering IMFs automatically. Finally the noise was suppressed significantly by both VWG and VGW methods.

### Noise Reduction Effect on Crying Noise

**Figure 5:**
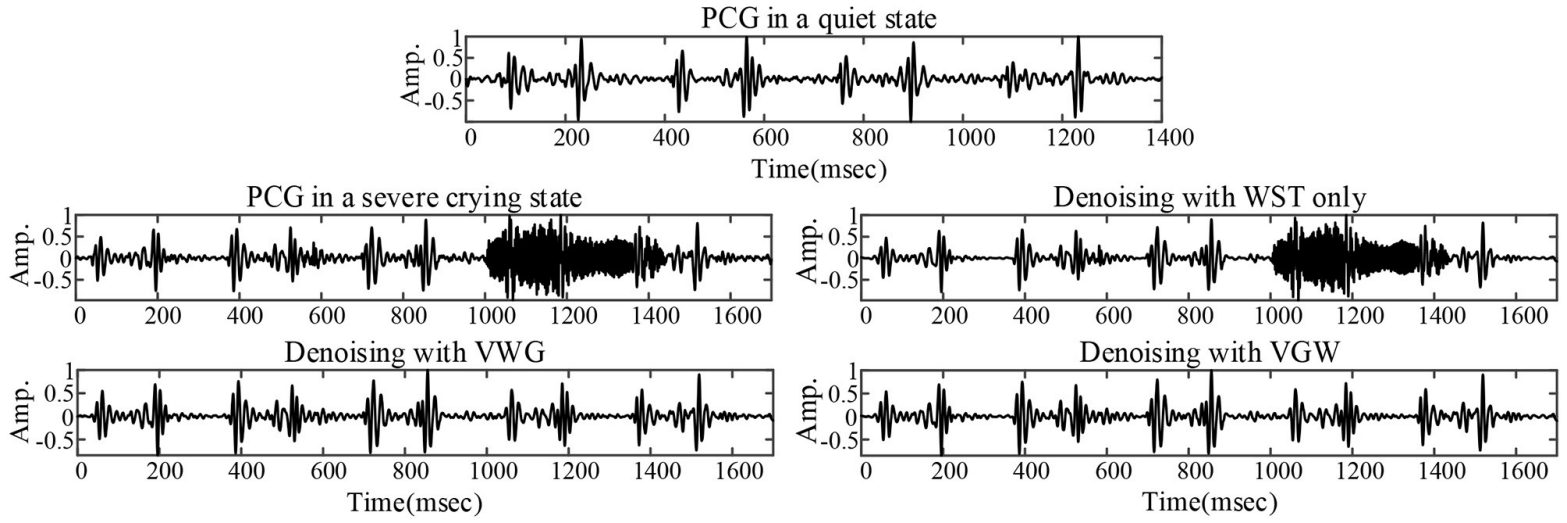
PCG without crying noise (top), PCG with severe crying noise and noise reductions for the PCG with severe crying noise.

**Figure 6:**
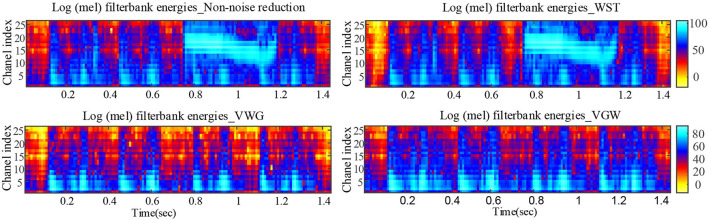
Comparison of Mel filter energies after noise reductions for PCG with severe crying noise.

The PCG of a child in a quiet state is shown in Figure 5, mainly with systolic murmurs. The noise due to the loud crying was in the fourth cardiac cycle and almost dominated the heart sound wave form in the cycle shown in Figure 5. Figures 5, 6 compared the denoising performance of the WST, VWG, and VGW for the PCG including crying noise from the time domain and the time-frequency domain, respectively. As shown in Figure 6, the impact of these were crying noise was mainly manifested in the energy of the 9th and above filters, which mixes the PCG components above 155 Hz. It can be seen that the burst noise was filtered out with the systolic murmurs preserved, but the WST method did not suppress the crying noise. Correspondingly, the *dnSNRs* obtained by using the WST, VWG, and VGW to denoise the loud crying noise are 15.93, 3.48, and 3.49 dB, respectively. And the *dnSNRs* obtained by using these methods to denoise the 8 fragments randomly cut from the PCG of 40 s were 14.22, 10.91, and 11.03 dB, respectively. The *dnSNRs* obtained by the proposed denoising method were significantly lower than those by the WST only.

### Diagnosis After Noise Reduction

**Figure 7:**
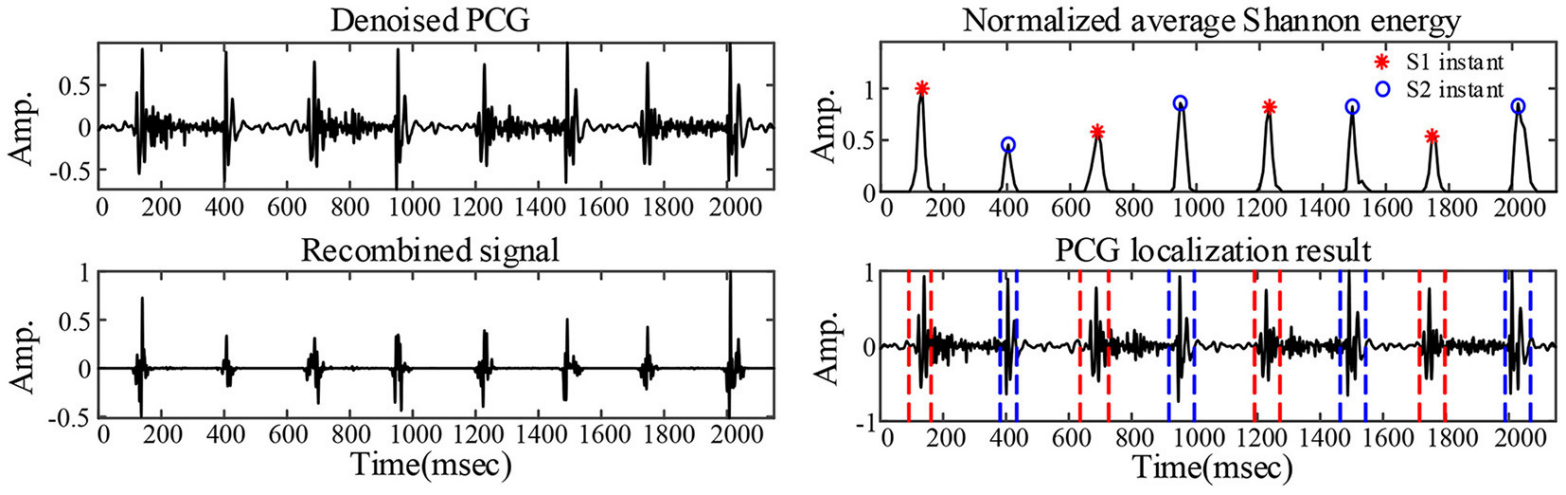
PCG segmentation after noise reduction.

**Figure 8:**
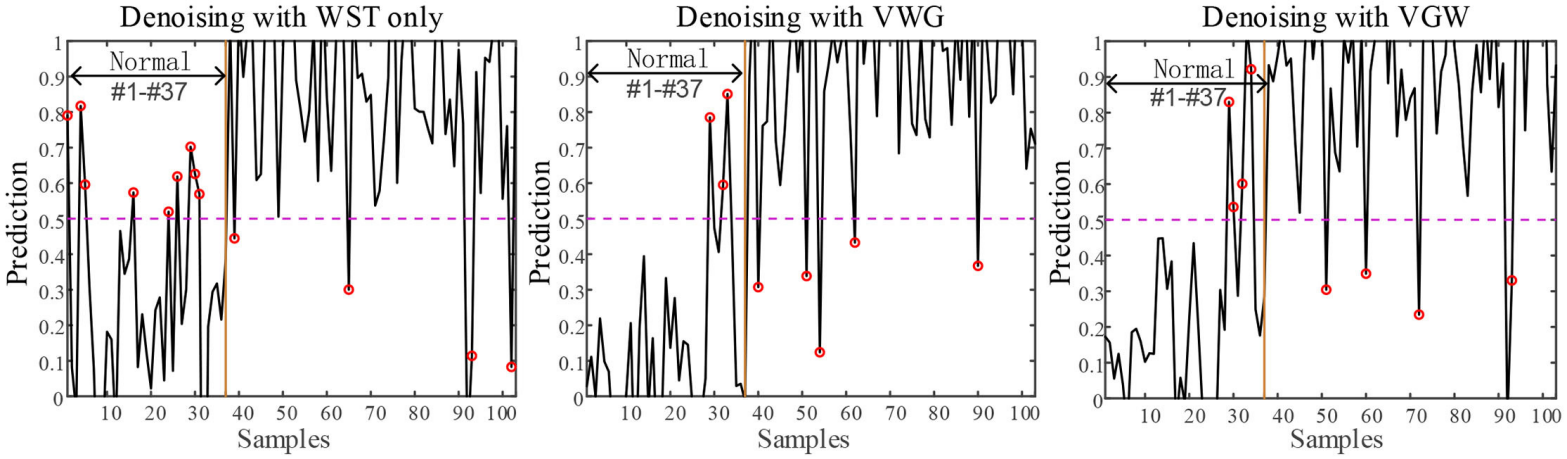
The results of PCG classification with the WST only, VWG and VGW, respectively.

An example of the segmentation of the PCG of a 1-year-old child with the ventricular septal defect denoised with the VWG is shown in Figure 7. The predictions obtained from the training of the features extracted after using the 3 noise reduction methods are shown in Figure 8. In the classification using the WST only, 9 cases with the normal heart were misclassified and 4 cases with abnormal heart were misdiagnosed. In the classification using the VWG, 3 cases in the normal heart falsely predicted and 5 cases in the abnormal heart falsely predicted. In the classification using the VGW, 4 cases in the normal heart falsely predicted and 4 cases in the abnormal heart falsely predicted. The accuracy, sensitivity, and specificity of the intelligent classifications are shown in Table 4. The classification using the VWG achieved the best performance but with no improved sensitivity.

**Table 4:** Classification performance based on different noise reduction methods:accuracy (Acc), sensitivity (Se), and specificity (Sp).

**Methods**	**Performance**		
	**Acc**	**Se**	**Sp**
WST only (14)	87.38%	93.94%	75.68%
VWG	92.23%	92.42%	91.89%
VGW	92.23%	93.94%	89.19%

## Discussion

This study proposed a noise reduction method combining both the VMD and WST according to the random and diverse characteristics of noise. The method is adaptive in decomposition and the selection of IMFs. The VMD and WST complement each other and optimize denoising effect on PCG by suppressing strong both the Gaussian noise and burst noise. It is found that the method can effectively suppress the noise in PCG, improve the performance of intelligent screening for congenital heart disease.

### Advantages and Shortcomings

Although the wavelet threshold method is widely employed for pre-processing of PCG classification, the threshold function affects approximation of the denoised signal to the original signal. The WST lacks adaptability to suppress the burst noise in PCGs because the wavelet function and threshold function have been set before the noise reduction, which was proved by the denoising effect on crying noise in the current study (Figures 5, 6). According to Figure 6, the deficiency of the WST also compromised the performance of the classification based on the Mel-frequency cepstral coefficients. The denoised PCGs with the WST (14) were smooth, but lost some details in the heart sound (marked by blue arrows in Figure 3), which may be the reason for the lower signal-to-noise ratios obtained by the WST on the reduction of the ambient noise (Table 2). VMD separates noise and heart sounds by decomposing them into different modalities. However, when the number of decomposition layers is fixed, the decomposition performance decreases as the noise increased, resulting in the noise and heart sound components in the same IMF. Thus, improper screening of IMFs can lead to the residual noise or loss of heart sound components. The waveform distortion of the second heart sound occurs in Figure 3 because only the last IMF was selected after decomposition when the major heart sound components were present in both of the last 2 IMFs. The similar reason is given for the worse denoising metrics obtained by the VMD-based method (29) for Gaussian noise with a signal-to-noise ratio of 10 dB in Tables 2, 3. The residual noise in the last IMF after decomposition leads to several poor denoising metrics obtained by the VMD-based method for Gaussian noise with a signal-to-noise ratio of 5 dB. To address these weaknesses, this study proposes the combination of the VMD and WST for PCG noise reduction. The adaptability of VMD provides a theoretical basis for the separation of noise from heart sounds, especially burst noise. The *pec*_*k*_ and *RSig*_*k*_ of IMFs were employed to compare the heart sounds before noise reduction to screen the IMFs, which retains IMFs with as many heart sound components as possible and as little noise as possible for reducing signal distortion. Therefore, the VWG and VGW are also adaptive in the screening of IMFs. As shown in Figure 4, after the PCG was decomposed into the 6 IMFs by VMD, the noise was separated into the first and second IMFs, which were automatically picked out and rejected due to their each *RSp*_*k*_>0.6 and *RSig*_*k*_ <0.5. However, some of the IMFs used for reconstruction still contain residual noise. So, the WST was employed for further noise reduction. The results proved that the VWG and VGW outperform the WST and the VMD-based method (29).

The current noise reduction algorithm has to be optimized in parameter settings. The denoising performance still degrades at high levels of noise. For example, the mean signal-to-noise ratio obtained by the current denoising method was reduced by 18% at 5 dB Gaussian noise. In addition, although the current noise reduction method improved the performance of the intelligent diagnostic system, there were still several cases of the normal PCG which were misclassified as abnormal. Continuous low-frequency noise was present in these recordings and resulted in high correlation coefficients between noise-containing IMFs and non-noise-reduced PCG, which affected the screening of IMFs. We tried to adjust parameters in the current denoising method for further improvement of noise reduction and found it to be feasible. Therefore, adaptive adjustment of the noise reduction parameters in the method was needed so that the method can cope with more types of noise in PCGs.

Furthermore, it should be noted that the features employed for diagnosis are not sufficient to distinguish mild murmurs from the normal heart sound. The current noise reduction method failed so that 4 congenital heart disease murmurs were classified as the normal heart sounds. These murmurs were weak and cloud easily be classified as the normal heart sounds. The degree of murmurs also need to be considered in intelligent diagnosis and the features that better characterize mild murmurs vs. the normal heart sounds need to be explored.

### Performance Comparison

The performance of the current methods was compared with those of the previous studies in PCG noise reduction or classification, as shown in Table 5. The signal-to-noise ratios obtained in the current study were only lower than those of studies (17) and (33). Both studies are blind source separation methods that could effectively separate S1 and S2 from the PCG with noise, and eliminate murmurs. Although blind source separation methods obtained high signal-to-noise ratios, the murmurs for classification were ignored. The difference in the databases used in the studies was also the reason for the significant difference in the signal-to-noise ratios obtained by each method, such as study (29). In study (29), there was a lack of adaptability in modal screening and the set of *K* of VMD was not based on analysis of the spectrum of the heart sounds, which might cause under-decomposition. From the studies using the same dataset, the current noise reduction method performed best in terms of the signal-to-noise ratio. It is 1-sided to consider only noise reduction metrics such as signal-to-noise ratio for the intelligent diagnosis studies, and more important to consider the retention of valid information such as murmurs. The deep learning approach performs best for heart sound classification, but its development for congenital heart disease screening is limited by the dataset size of children's PCGs. Performance of the methods in all the studies was above 90% except the classification using the WST for PCGs denoising. In general, the classification based on the proposed denoising method has comparable performance in diagnosing congenital heart disease murmurs.

**Table 5:** Comparison of performance with other methods.

**Method**	**Database**	**Performance**
WPT and SVD (33)	/	SNR:22.21 dB (10 dB), 18.37 dB (5 dB)
VMD denoising (29)	PCGs collected clinically (Michigan)	SNR:24.1 dB (10 dB), 19.1 dB (5 dB)
	Ours	SNR:7.99 dB (5 dB)
GSD (17)	PCGs collected clinically (Michigan)	SNR:30.3 dB (10 dB), 35.26 dB (15 dB)
OMLSA and WT (34)	PCGs collected clinically (Washington)	SNR:11.76 dB (5 dB)
Matched Filters, Support Vector Machine, ANN (35)	PCGs collected clinically	Se = 84–93%,Sp = 91–99%
Wavelet hard thresholding, iterative backward elimination, SVM (11)	PCGs collected clinically	Acc = 92.6%
Butterworth band-pass filter, MFCCs, CRNN (12)	The CinC challenge 2016 database	Se = 98.66%, Sp = 98.01%, Acc = 98.34%
WST (14), ANN	Ours	SNR:10.64 dB (10 dB), 7.56 dB (5 dB) Acc = 87.38%, Se = 93.94%, Sp = 75.68%
Proposed, ANN	Ours	SNR:14.91 dB (10 dB), 12.39 dB (5 dB) Acc = 92.23%, Se = 92.42%, Sp = 91.89%

### Limitations

Only 1 case with the crying noise was employed for the experiment to show effect of the method on suppression of the crying noise. However, prediction of intelligent diagnosis was performed with noise reduction in all the data. The effectiveness of the noise reduction method for the adult heart sounds is not studied in detail in this study. The PCG samples of children's heart sounds suffered from low quantities and data imbalance.

## Conclusion

A novel denoising method based on combined VMD and WST is proposed in the study. This method allows for flexibility in the selection of IMFs in VMD noise reduction and overcomes the short comings of denoising strong Gaussian noise with VMD and suppressing burst noise with WST. The method results in better noise reduction metrics than previous methods and effectively suppresses the noise in the child PCG from the congenital heart disease, but not the murmurs. It is found that the performance of classification system based on VWG method is improved and the accuracy of intelligent diagnosis of the congenital heart diseases is enhanced with the PCG denoised by the method.

## Data Availability Statement

The raw data supporting the conclusions of this article will be made available by the authors, without undue reservation.

## Ethics Statement

Ethical review and approval was not required for the study on human participants in accordance with the local legislation and institutional requirements. Written informed consent to participate in this study was provided by the participants' legal guardian/next of kin.

## Author Contributions

AZ and JW designed a protocol for intelligent screening for precardiac disease. AZ proposed the noise reduction method and designed the experiments while drafting the manuscript. JW collected the dataset and assisted in the code writing of the intelligent diagnosis part. FQ provided part of the PCG samples and the electronic stethoscopes. ZH supervised the research project, was responsible for the overall direction and provided insights, and revised the manuscript. All authors contributed to the article and approved the submitted version.

## Conflict of Interest

FQ is employed by Shanghai Lishen Information Technology Co., Ltd. The remaining authors declare that the research was conducted in the absence of any commercial or financial relationships that could be construed as a potential conflict of interest.

## Publisher's Note

All claims expressed in this article are solely those of the authors and do not necessarily represent those of their affiliated organizations, or those of the publisher, the editors and the reviewers. Any product that may be evaluated in this article, or claim that may be made by its manufacturer, is not guaranteed or endorsed by the publisher.
